# Emotional Stress and Immune Response in Surgery: A Psychoneuroimmunological Perspective

**DOI:** 10.7759/cureus.48727

**Published:** 2023-11-13

**Authors:** Taufiqa Reza, Han Grezenko, Chad Barker, Danyal Bakht, Nuzhat Faran, Noor Abdullah Yahya, Maryam Affaf, Hana Mohamed, Rayan Gasim, Mohammed Khaleel I.K.H. Almadhoun, Abdur Rehman, Uday Kumar, Abdullah Shehryar, Abdul Haseeb Hasan

**Affiliations:** 1 Medicine, Avalon University School of Medicine, Youngstown, USA; 2 Medicine and Surgery, Guangxi Medical University, Nanning, CHN; 3 Translational Neuroscience, Barrow Neurological Institute, Phoenix, USA; 4 Department of Public Health, University of South Florida, Tampa, USA; 5 Medicine and Surgery, Mayo Hospital, Lahore, PAK; 6 Internal Medicine, Fatima Memorial Hospital, Lahore, PAK; 7 Family Medicine, Dubai Medical College, Dubai, ARE; 8 Internal Medicine, Women’s Medical and Dental College, Abbottabad, PAK; 9 Medicine, United Nation Study & Understanding, The International Academy, Khartoum, SDN; 10 Medicine, Elrazi University, Khartoum, SDN; 11 Internal Medicine, University of Khartoum, Khartoum, SDN; 12 Medicine and Surgery, Mutah University, Karak, JOR; 13 Surgery, Mayo Hospital, Lahore, PAK; 14 Medical School, Shaheed Mohtarma Benazir Bhutto Medical College Lyari, Karachi, PAK; 15 Internal Medicine, Allama Iqbal Medical College, Lahore, PAK; 16 Internal Medicine, Mayo Hospital, Lahore, PAK

**Keywords:** psychological factors, surgical outcomes, operating room, psychoneuroimmunology, surgery, immune response, emotional stress

## Abstract

Psychoneuroimmunology (PNI) offers a deep dive into the nexus between emotional stress, immunity, and surgical outcomes. In this narrative review, we first trace PNI’s historical roots, providing a foundational understanding of its evolution. We then dissect its significance across the surgical journey, from the preoperative phase through to postoperative recovery.

It becomes evident through our exploration that emotional stress has profound implications for surgery, notably influencing wound healing rates, susceptibility to infections, and overall postoperative well-being. Among the arsenal to combat these challenges, interventions such as cognitive-behavioral therapy, mindfulness, and complementary practices such as meditation and yoga have emerged as potent tools. They not only mitigate stress but also play a pivotal role in enhancing immune function.

However, the journey to optimizing surgical outcomes is not just about identifying effective interventions. A resounding theme is the importance of holistic care, ensuring that all patients have equitable access to these tools. As PNI continues to evolve, we stand at the precipice of a healthcare revolution, one that promises a blend of personalized care, anchored in a deep understanding of the mind-body connection in surgical contexts.

## Introduction and background

Psychoneuroimmunology (PNI) is the investigation of alterations in immunity connected to behavior and changes in behavior linked to immunological responses stemming from the interactions among the nervous, endocrine, and immune systems [1]. It offers a comprehensive bio-psycho-social perspective on health and illnesses, challenging the conventional perception of the immune system as an autonomous entity. PNI has emerged as a significant advancement in comprehending the interaction between psychosocial variables, health, and illness by examining behavioral changes linked to immunity and immunological changes associated with behavior [2].

Substantial research within the realm of PNI elucidates the profound influence of emotions on health. Many studies have delineated the association between adverse emotional states and heightened mortality risks [3]. In contrast, a state of robust psychological well-being is linked to reduced mortality rates [4]. The intricate correlation between mood and the immune or inflammatory response underscores that enhanced and suppressed immune reactions can lead to unfavorable clinical outcomes [2]. This accentuates the importance of a nuanced understanding of the myriad behavioral aspects, including psychological states, in influencing health.

Furthermore, PNI has expounded on the impact of stressors on health outcomes. Although acute stress reactions are generally adaptive, prolonged stress, particularly in elderly or vulnerable individuals, can yield detrimental effects. The nature and magnitude of stressors, biological variables, psychosocial resources, and coping mechanisms collectively influence how stress impacts health. Significantly, psychosocial interventions have demonstrated efficacy in managing stress-related disorders and potentially altering the trajectory of chronic conditions [5]. Thus, PNI enriches our comprehension of the mind-body connection and provides invaluable insights into healthcare and disease management.

Surgery often induces anxiety and fear in patients awaiting their procedures. Studies have shown that 60-80% of individuals experience preoperative anxiety, significantly affecting postoperative aspects such as wound healing, pain control, anesthesia efficacy, and analgesia needs. Additionally, a patient’s emotional state before surgery can influence their physical and psychological recovery afterward [6]. New findings emphasize the significant influence of psychological factors on surgical outcomes, spanning both immediate and lasting effects [7]. Thus, it is essential to pinpoint the factors contributing to fear and anxiety and uncover their relationships. This understanding is vital for crafting personalized interventions to improve surgical experience and postoperative healing [6].

This comprehensive narrative review explores the intricate interplay between psychology, immunology, and surgery. It traces the historical evolution of PNI, the scientific domain that bridges emotional and psychological states with immune function. The review scrutinizes the theoretical frameworks that underlie this discipline, providing in-depth insights into the mechanisms involving neurotransmitters, cytokines, and hormonal pathways. Additionally, it underscores the impact of emotional stress on the preoperative, perioperative, and postoperative phases of surgery, encompassing facets such as immune suppression, infection susceptibility, and recovery duration. The narrative also delves into medical interventions designed to alleviate stress, including psychological counseling and pharmacological options. Limitations, ethical considerations, and future research directions are addressed, presenting a comprehensive perspective on how PNI shapes surgical outcomes.

## Review

Historical perspective

The genesis of PNI can be traced back to antiquity when scholars across diverse cultures pondered over the intricate interplay between mental states and physical well-being. Despite this, it was not until 1975 that the structured investigation into the relationship between the nervous, endocrine, and immune systems was formally acknowledged. This milestone was achieved thanks to Robert Ader, who introduced the term “psychoneuroimmunology” [8]. Pioneering studies that connected psychology with surgery laid the foundation for this field by acknowledging the role of psychological variables in postsurgical recuperation. It was recognized that psychological factors, including emotions, stress, and depression, influenced wound healing, consequently impacting surgical outcomes [9]. Beyond direct pathways involving stress hormones, more indirect factors, such as the selection of anesthetic agents and the preoperative health status, were discerned to be susceptible to psychological states [10].

Immunology entered the discourse when scientists explored the physiological mechanisms through which psychology could influence the immune functions of the human body. This pivotal moment heralded the inception of PNI as a field [9]. Research in this arena has unequivocally demonstrated that surgery and pain can unfavorably affect immune function. Pain, stress, and depression were identified as psychoneurological phenomena capable of impacting immune function through neuroendocrine pathways. Consequently, the planning and administration of anesthesia care have increasingly incorporated the assessment and management of postoperative pain to ameliorate immune function and overall surgical outcomes [11].

The Crucial Role of Psychosocial Factors in Surgical Outcomes

Recent revelations in PNI highlight the profound role of psychosocial factors, such as attitudes and moods, in determining surgical outcomes. These psychological elements can often overshadow even clinical factors in their predictive capacity. For instance, a patient’s mental resilience or underlying stressors can greatly influence their rate of recovery and susceptibility to postoperative complications. Understanding a patient’s psychological profile is thus pivotal, informing surgeons not just about the surgical procedure but also about preoperative preparations and postoperative care strategies. Recognizing potential anxiety or stress allows medical teams to deploy tailored interventions, ranging from counseling to pharmacological aids, optimizing surgical outcomes. This holistic approach underscores the collaborative essence of healthcare, where surgeons, considering both physiological and psychological needs, engage in meaningful dialogue with patients, fostering tailored and comprehensive recovery strategies [12].

Theoretical frameworks

Situated at the crossroads of psychology, neuroscience, and immunology, the multifaceted discipline of PNI serves as a beacon for understanding the intricate interplay among the behavioral, neurological, and endocrine systems and their collective influence on the immune response within humans [13]. Rooted in its foundational principles is the revolutionary idea that the immune system does not operate in isolation. Instead, it functions as a part of a larger, dynamic feedback mechanism, continuously interacting with and being influenced by the central nervous system (CNS). This perspective challenges traditional views, positioning the immune system not merely as a defender against external pathogens but as an active participant in our body’s holistic health dialogue. As researchers delve deeper into the molecular and cellular intricacies binding these systems, a revelation emerges: a piecemeal approach is inadequate. To truly appreciate the depth and breadth of these connections, there is an urgent need for a comprehensive strategy that considers not just the physiological but also the cognitive and emotional facets of the human experience [14]. This broadened perspective promises not only a richer understanding of health and disease but also the potential for more integrative and holistic therapeutic approaches in the future.

Emotional stress, an omnipresent facet of daily existence, potently influences immune function. Stress infiltrates the body through multiple avenues. First, sympathetic nerve fibers extend from the cerebral cortex into primary and secondary lymphoid tissues, releasing bioactive substances that engage with immune cells, and modulate their reactivity [15]. Second, the sympathetic-adrenal-medullary and hypothalamic-pituitary-adrenal (HPA) axes generate hormones and bioactive peptides, including cortisol and adrenaline, influencing leukocytes [16]. Third, individuals grappling with stress may manifest behavioral adaptations, such as alterations in sleep patterns or increased alcohol consumption, with the capacity to modify processes within the immune system, thus establishing an additional conduit uniting stress and immunological responses [17].

Neuroimmune Crosstalk: Significance of Soluble Cytokines and Neuronal Pathways in Bidirectional Communication

Soluble cytokines (interleukin (IL)-6, IL-1, and tumor necrosis factor (TNF)-α) and neuronal pathways (HPA axis and the sympathetic nervous system (SNS)) facilitate bidirectional communication between the immune system and the CNS. The activation of these two neurochemical pathways and the subsequent release of hormones and neurotransmitters can exert significant downstream impacts on immune function. It is worth noting that each of the hormones and neurotransmitters released from these nervous system regions has demonstrated the potential, in both in vivo and in vitro settings, to influence various aspects of the immune system [18]. The expression of proteins and messenger RNA for neurologically relevant hormones, particularly within T cells, underscores the interconnectedness of psychological and immunological processes [19,20].

**Figure 1:**
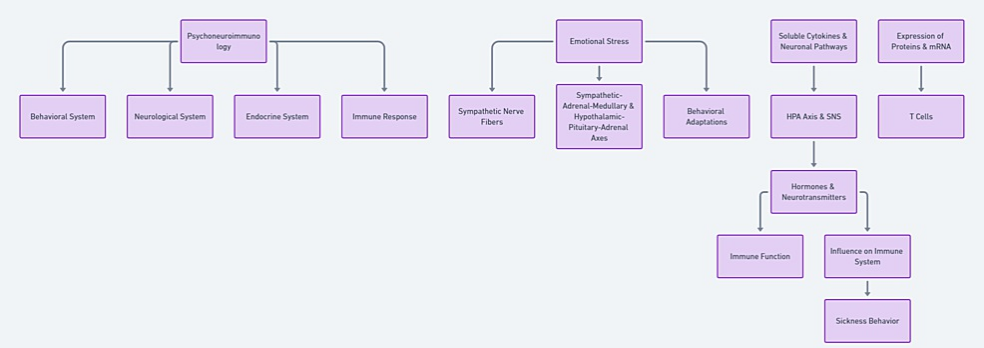
Interplay of psychoneuroimmunology: A holistic view of the influence of behavioral, neurological, and endocrine systems on immune response. This schematic illustrates the multifaceted connections between emotional stress, the central nervous system, and the immune system, emphasizing the bidirectional communication pathways and the profound impact of psychological factors on immune functionality. The image is generated by the authors.

Notably, the parallels between the relationship of stress and the immune system to the immune response to infection, typified by the syndrome of sickness behavior, are noteworthy [21]. This complex of symptoms encompasses diminished physical activity, reduced inclination for social interaction, decreased appetite, heightened pain sensitivity, and emotional despondency. These congruent responses underscore the organism’s adaptive capacity to judiciously allocate resources during periods of physiological challenge [15]. A summary of the entire discussion has been presented in schematic form in Figure 1.

Further insights into the interactions between the mind, brain, and immune system have been provided by Whitesman and Booth, who highlight the importance of understanding the common molecular language shared by the neuroendocrine and immune systems, which integrates the two primary systems involved in systemic responses [22]. Additionally, the role of PNI in personalized and systems medicine has been emphasized by Yan, who discusses how PNI might provide the scientific foundation for personalized and systems medicine, emphasizing the exploration of interactions among psychological, behavioral, nervous, immune, and endocrine systems to understand health, wellness, and diseases [23].

Role of emotional stress in the preoperative and perioperative phases

PNI plays a significant role in understanding the complex relationship between emotional stress and the preoperative and perioperative phases of medical procedures. It is typical for patients awaiting surgical procedures to experience preoperative anxiety and stress. Numerous physiological systems, including the release of hormones and neurotransmitters such as cortisol, adrenaline, and norepinephrine, control responses to stress in humans. Elevated heart rate and blood pressure, which are signs that the SNS is working, are the results of these reactions [24]. Notably, perioperative physiological changes leading to diseases such as tachycardia and hypertension are indicators of emotional anxiety and stress in surgical patients [25,26].

The management of preoperative stress and anxiety is of paramount importance in the surgical journey, directly influencing not just the patient’s mental well-being but also tangible clinical outcomes. A significant proportion of patients, i.e., around 60% to 80%, in Western populations face heightened levels of anxiety as they approach their surgical procedures [27]. While it is easy to dismiss these feelings as natural or expected, the repercussions of such anxiety are profound and multifaceted.

Beyond the immediate psychological distress, heightened anxiety alters physiological responses, leading to tangible changes in how patients respond to medical interventions during surgery. For instance, their physiological state, influenced by their mental stress, may necessitate alterations in anesthesia administration, with anxious patients often requiring higher dosages to achieve the same effect. This increased need can pose additional risks and complications, particularly given the delicate balance required in anesthesia. Moreover, a heightened requirement for analgesics postsurgery may also emerge, aiming to manage pain exacerbated by preoperative anxiety. This, in turn, can result in prolonged hospital stays, increased medical costs, and potentially, a longer road to full recovery.

Furthermore, anxiety and depression have been correlated with adverse perioperative outcomes. Dysregulation of the HPA axis, a central stress response component, plays a pivotal role in these conditions. In instances such as depression, impaired glucocorticoid receptor functioning at the limbic-hypothalamic level can result in glucocorticoid resistance and heightened production of stress hormones. Such dysregulation may render patients more susceptible to infections and impede wound healing, ultimately impacting surgical outcomes [28].

**Figure 2:**
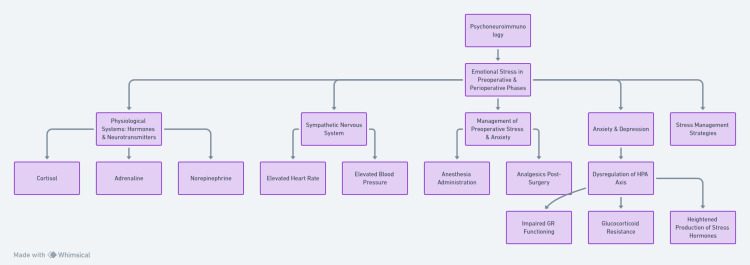
An overview of the interplay between emotional stress and physiological responses in the preoperative and perioperative phases. The image is generated by the authors.

Recognizing this pivotal juncture, researchers have explored stress management strategies to mitigate the psychological, physiological, and immunological repercussions of preoperative stress. Clinical investigations have unveiled that this “window of opportunity” before surgery can be harnessed to ameliorate stress responses, potentially enhancing immune function and reducing the risks of potential complications [29]. An overview of this interplay has been illustrated in Figure 2.

Role of emotional stress in the postoperative phase

Emotional stress is pivotal in the postoperative phase, significantly affecting multiple facets of a patient’s recovery. One critical dimension where stress exerts its influence is wound healing. The research underscores a clear and clinically meaningful correlation between psychological stress and the healing of surgical wounds. Elevated stress levels have been associated with protracted wound repair, amplifying the risk of complications, including infections [30]. For instance, patients reporting heightened stress before surgery are inclined to experience prolonged hospitalizations, an elevated incidence of postoperative complications, and heightened rehospitalization rates [10,12]. Conversely, optimism has been revealed to have a protective influence, with more optimistic patients evidencing reduced rehospitalization rates and enhanced healing [31]. Moreover, individuals manifesting depressive symptoms are more likely to encounter postoperative infections and impaired wound healing [32].

Furthermore, the impact of emotional stress extends beyond surgical wounds to chronic wounds, with patients manifesting high levels of depression and anxiety being fourfold more likely to belong to the delayed healing cohort [33]. Remarkably, these effects remain conspicuous even after adjusting for sociodemographic variables and medical conditions [30].

*Emotional Stress*’*s Impact on Postoperative Quality of Life*

The repercussions of emotional stress are not confined to wound healing but extend to health-related quality of life (HRQoL) in the postoperative period. Major surgical procedures instigate a metabolic stress response and inflammation, engendering symptoms such as fatigue, diminished motivation, and withdrawal from routine social activities, collectively recognized as sickness behavior. Patients frequently anticipate a swift amelioration in their HRQoL following surgery, potentially underestimating the discomfort they may encounter in the initial postoperative period. This discordance can negatively impact short-term postoperative HRQoL, amplifying the risk of complications and discomfort during recuperation [34].

Consequently, the vigilance and addressing of emotional stress in the postoperative phase should form an integral component of perioperative care [35]. By recognizing and managing stress, healthcare professionals can augment wound healing, reduce infection susceptibility, shorten hospitalizations, and ultimately enhance patients’ overall quality of life after surgery.

Medical interventions to mitigate stress and enhance immunity

In recent years, there has been a heightened emphasis on medical interventions tailored to counteract stress and bolster immunity, particularly given the ever-increasing understanding of the profound linkage between mental well-being and physiological health. It is no longer merely an academic assertion; stress, with its manifold repercussions, has been unequivocally identified as a factor that can weaken the immune system, leaving individuals more susceptible to diseases and reducing their overall vitality [36].

This recognition has catalyzed a flurry of research and development, leading to the formulation of a suite of psychological interventions designed to alleviate stress. Techniques such as cognitive-behavioral therapy delve into thought patterns and behaviors, guiding individuals to develop more adaptive reactions to stressors. Mindfulness practices, rooted in ancient traditions but modernized for contemporary application, teach individuals to stay present and cultivate a non-judgmental awareness of their experiences. This can bring about a profound calm and balance in the face of life’s adversities. Similarly, relaxation techniques, varying from progressive muscle relaxation to deep breathing exercises, provide individuals with tools to consciously dial down their stress levels [36].

Exploring Mind-Body Medicine: Holistic Approaches to Health

Mind-body medicine, a prominent category of complementary and alternative medicine, has gained prominence, notably in the United States [37]. This field centers on the interplay among the brain, mind, body, and behavior and their collective influence on health and disease. Modalities such as hypnosis, meditation, yoga, and tai chi have been associated with a range of positive outcomes, encompassing the alleviation of conditions such as chronic pain, headaches, and mood enhancement, along with the improvement of quality of life [38]. These modalities have also effectively managed symptoms associated with various diseases and treatments, including chemotherapy-induced nausea and pain in cancer patients [39]. Their appeal stems from their minimal physical and emotional risks, cost-effectiveness, and the empowerment of patients to assume an active role in their treatment [38].

To address anxiety, psychotherapy has emerged as a preference over psychotropic medications. While the incorporation of cognitive-behavioral interventions with medication has been explored, its effectiveness varies and is generally not regarded as a primary approach. Nonetheless, a combined treatment approach may be considered for individuals contending with severe anxiety necessitating medication [40]. These findings underscore the diverse range of medical interventions available for addressing stress and its implications for immune health, empowering individuals to make informed decisions for their well-being.

Limitations and ethical considerations

Considering surgery as a psychological stressor brings forth three main concerns. To begin, various recovery indicators often lack alignment, with some lacking clinical clarity and others, such as hormonal changes, holding promise for insights. Second, the impact of psychological factors on recovery remains unclear due to disparities between physiological and psychological findings. Finally, interventions aimed at modifying preoperative emotional states or coping approaches yield varying outcomes with uncertain mechanisms [41].

Navigating Ethical Challenges in Informed Consent for Psychotherapy

Securing genuine informed consent in psychotherapy presents an intricate ethical challenge. The imperative lies in revealing potential treatment side effects while upholding principles of autonomy and nonmaleficence. Paradoxically, this transparency may trigger nocebo effects, where negative expectations result in adverse consequences [42,43]. Patients informed from the outset about potential side effects are more prone to experience them. Regrettably, clinical informed consent often fails to meet legal and ethical benchmarks, disregarding patients’ informational and decision-making demands. Given the diverse needs of individuals with mental disorders, it is imperative to adopt a personalized approach to consent information, departing from a one-size-fits-all model [44].

Prioritizing Equity in Access to Holistic Healthcare

Equity in access to holistic care assumes paramount significance. Ideally, everyone should enjoy equal opportunities for optimal health, and disparities should be preventable. Health inequalities are influenced by socioeconomic determinants across a person’s life course, stemming from government policies and resource distribution. Effectively addressing these disparities necessitates adopting a holistic perspective on health inequalities, accounting for the broader societal factors contributing to health inequities [45].

An alternative strategy centers on factors within the surgical ward, such as social support and coping opportunities, which can impact stress responses. Nonetheless, these approaches necessitate thorough evaluation for both their physiological and psychological effects before becoming standard practice [41].

Future directions

Future directions in PNI hold the promise of ushering in exhilarating advancements in our understanding of the intricate links between psychological factors, the nervous system, and the immune system. The field of PNI is still evolving, with many unanswered questions awaiting comprehensive exploration. A principal concern centers on the magnitude and consistency of the association between attachment, emotions, and the immune system. As more studies come to fruition, researchers aspire to establish direct connections between attachment orientation and immune metrics [46].

The holistic approach of PNI, scrutinizing the interplay among psychological, neurological, and immunological elements, bears the potential to advance personalized medicine. By discerning individual psychological and physiological profiles, PNI can contribute to tailoring healthcare to align with the distinct needs of patients. This is especially pertinent, given the links between stress, inflammation, and various health conditions. Insights from PNI may catalyze the identification of systemic therapeutic targets, subsequently informing personalized treatment strategies spanning drug therapies, dietary regimens, supplements, and mind-body interventions [47].

Technological advancements, including wearables and mobile applications, assume a pivotal role in the future of PNI. These tools enable the continuous monitoring of stress levels and other biomarkers, providing a comprehensive perspective of an individual’s well-being. By harnessing these technological aids, healthcare professionals can more effectively assess and manage stress and distress [47-49]. Additionally, biofeedback training, which has showcased promise in diminishing anxiety and enhancing self-regulation, can be more integrally woven into these monitoring systems, thereby enhancing their utility [47].

As the sphere of PNI advances, it has the potential to redefine our approach to health, well-being, and disease prevention by bridging the chasm between the mind and the body. These future trajectories augur more precise, personalized healthcare solutions and more profound recognition of how our emotions and mental states modulate our physical health. This progress in PNI will empower healthcare professionals to deliver more efficacious care and propagate overall well-being on an individualized basis [50].

## Conclusions

PNI delves deep into the intertwined relationships between the mind, nervous system, and immune response, challenging the traditional notion of an isolated immune system. Tracing its historical evolution, PNI highlights the crucial role of psychological elements in determining surgical outcomes. The complex frameworks within PNI shed light on how emotional stress, mediated by a cocktail of hormones, neurotransmitters, and cytokines, directly influences immune functionality.

Emotional stresses, particularly evident during surgical processes, impact vital recovery aspects such as wound healing and postoperative well-being. This necessitates a proactive role by healthcare professionals in stress management to optimize surgical results. While interventions such as cognitive-behavioral therapy offer promise, challenges in ethical consent and health disparities remain. The future of PNI, however, is optimistic, pointing toward a meld of personalized medicine, technological innovation, and a revamped holistic approach to health.
